# Investigation of Risk Factors Related to the Development of Hepatic Dysfunction in Patients with a Low and Moderate Cardiac Risk During Open-Heart Surgeries

**DOI:** 10.21470/1678-9741-2019-0427

**Published:** 2021

**Authors:** Ayse Baysal, Gonul Sagiroglu, Mevlut Dogukan, Ismail Ozkaynak

**Affiliations:** 1 Pendik Bolge Hospital, Department of Anesthesiology and Reanimation, Istanbul, Turkey.; 2 Department of Anaesthesiology and Reanimation, Trakya University Medical Faculty, Edirne, Turkey.; 3 Department of Anaesthesiology and Reanimation, Adıyaman University Training and Research Hospital, Adıyaman, Turkey.; 4 Surreyyapasa Pulmonary Diseases and Thoracic Surgery Research and Training Hospital, Department of Anesthesiology and Reanimation, Istanbul, Turkey.

**Keywords:** Hyperbilirubinemia, Bilirubin, Aortic Valve, Cardiopulmonary Bypass, Potoperative Complications, Stroke Volume, Risk Factors

## Abstract

**Objective:**

To determine the possible risk factors associated with hepatic dysfunction during open-heart surgeries.

**Methods:**

After excluding 71 patients, 307 patients with possible low and moderate cardiac risk who underwent either coronary artery bypass graft surgery (CABG) (n=176) or valve repair surgery (mitral valve, mitral and aortic valves and/or tricuspid valve) (n=131) were investigated prospectively during a 6-month period. Hyperbilirubinemia is defined as an occurrence of a plasma total bilirubin concentration >34 µmol/L (2 mg/dL) in any measurement during the postoperative period; the patients were divided into groups with or without postoperative hyperbilirubinemia. The collected parameters were: alanine transaminase (ALT), aspartate transaminase (AST), alkaline phosphatase (ALP), total bilirubin (TBil), gamma-glutamyl transpeptidase (GGT) and albumin. The parameters were collected preoperatively and postoperatively on days 1, 3 and 7. Preoperative, intraoperative, and postoperative risk factors were investigated. Logistic regression analysis was performed to identify the risk factors for postoperative hyperbilirubinemia.

**Results:**

Postoperative hyperbilirubinemia was observed in 7 of 176 patients (4%) who underwent CABG, and in 11 of 131 patients (8.4%) who underwent valve replacement surgeries. Independent risk factors for early postoperative hyperbilirubinemia were found as: ejection fraction (EF), aortic cross-clamp (ACC) time, intensive care unit stay and extubation time (*P*<0.001). In comparison to CABG procedures, postoperative hyperbilirubinemia was observed more frequently in patients undergoing valve surgeries (*P*=0.027).

**Conclusion:**

Low EF and prolonged ACC time are significant independent risk factors for early postoperative hyperbilirubinemia during open-heart surgeries with cardiopulmonary bypass. Valve surgeries show a higher incidence of hyperbilirubinemia in comparison to CABG.

**Table:** 

Abbreviations, acronyms & symbols				
**ACC**	**= Aortic cross-clamping**		**GGT**	**= Gamma-glutamyl transpeptidase**
**ACT**	**= Activated clotting time**		**HR**	**= Heart rate**
**AF**	**= Atrial fibrilation**		**IABP**	**= Intra-aortic balloon pump**
**ALP**	**= Alkaline phosphatase**		**ICU**	**= Intensive care unit**
**ALT**	**= Alanine transaminase**		**LDH**	**= Lactate dehydrogenase**
**ANOVA**	**= Analysis of variance**		**NHYA**	**= New York Heart Association**
**ASA**	**= American Society of Anesthesiologists**		**PaO_2_**	**= Partial pressure of oxygen**
**AVR**	**= Aortic valve replacement**		**PaCO_2_**	**= Partial pressure of carbon dioxide**
**AST**	**= Aspartate transaminase**		**PRBCs**	**= Packed red blood cells**
**CABG**	**= Coronary artery bypass graft**		**SD**	**= Standard deviation**
**COPD**	**= Chronic obstructive pulmonary disease**		**SpO_2_**	**= Peripheral oxygen saturation**
**CPB**	**= Cardiopulmonary bypass**		**SPSS**	**= Statistical Package for the Social Sciences**
**CVP**	**= Central venous pressure**		**TBil**	**= Total bilirubin**
**EF**	**= Ejection fraction**			

## INTRODUCTION

There are a series of pathophysiological changes in patients undergoing open-heart surgeries with cardiopulmonary bypass (CPB) that cause liver hypoperfusion, centrilobular sinusoid ischemia, and subsequent reperfusion injuries, hemolysis, or systemic inflammatory response. These events may eventually lead to various forms of hepatic dysfunction in patients during the postoperative period after open-heart surgeries^[1]^. An increased incidence of liver function test abnormalities were reported, and rates vary between 10% and 40%^[2]^. The occurrence of postoperative hyperbilirubinemia is crucial during evaluation of factors associated with increased morbidity and mortality after open-heart surgery with CPB. There are several reports of possible risk factors associated with hepatic dysfunction and increased mortality^[3-5]^. In previous studies, the incidence of postoperative hyperbilirubinemia ranged from 20% to 51% in open-heart surgeries with CPB. The causes of this higher incidence were related to the presence of various possible risk factors, among them: valvular heart disease and low cardiac output causing hemodynamical instability, and low ejection fraction (EF). Other important risk factors for postoperative hepatic dysfunction after open-heart surgery with CPB were longer operative time and greater blood transfusion volume. However, CPB itself is not a significant constituent in the postoperative development of hyperbilirubinemia^[2,4]^. Splanchnic ischemia before or during the operation and in the postoperative period appears to be an essential cause^[2,5]^. Other risk factors were identified as possible risk factors for postoperative hepatic dysfunction. We can list these factors as: heart function with poor preoperative clinical and echocardiographic findings, hemodynamic instability, emergency surgery, and laboratory findings of poor preoperative liver dysfunction^[5-8]^.

The European System for Cardiac Risk Evaluation (EuroSCORE) was developed for risk assessment in open-heart surgeries to provide possible risk factors that may increase postoperative mortality and morbidity. The patients are usually divided into three risk groups using this scoring system and include: low-risk (0-2), medium-risk (3-5) and high-risk groups (≥6)^[9]^. In our study, we include patients with possible low and medium risk according to EuroSCORE preoperative cardiac risk scoring system and other possible cardiac risks. Our goal was to determine the possible risk factors associated with hepatic dysfunction in patients with low and moderate cardiac risk during open-heart surgeries with CPB.

## METHODS

Three hundred and forty consecutive patients undergoing open-heart surgery with CPB were studied in a prospective and observational study for nine months. The Ethical Committee of our institution have given approval for the study protocol. The study was registered at ClinicalTrials.Gov and the registration number is NCT04271098. Every patient or their caregivers gave written informed consent to enroll in the study. The ethical principles of the Declaration of Helsinki for human research ethics were followed.

Three hundred and seventy-eight patients were enrolled in the study. Fifty-three patients were considered for the study, however, they were excluded as they did not fulfill the inclusion criteria. Twelve patients refused to participate in the study. During the study, the study protocol was not completed in 16 patients and among them, five refused to give a blood sample during the study, and it was not possible to complete the records in 11 cases.

The inclusion criteria for the study were patients who underwent open-heart surgery with CPB, between 25 and 80 years of age, American Society of Anesthesiologists (ASA) status of 2 and 3, and preoperative EF >30%. The exclusion criteria include surgical procedure including both coronary artery bypass grafting (CABG) and valve replacement, resection of ventricular or aortic aneurysm, transplantation or another surgical procedure, reoperation of valve repair surgery, patients with preoperative EF <30%, preoperative hyperbilirubinemia defined as total bilirubin concentration >2 mg/dL, preoperative congestive heart failure causing severe restrictive changes in spirometric studies and these findings include: a reduction in vital capacity (VC), forced expiratory volume in 1 second (FEV_1_), and total lung capacity (TLC), preoperative chronic obstructive pulmonary disease (COPD) causing severe degree of changes in pulmonary function tests, including FEV_1_/forced vital capacity (FVC) <0.7 and FEV_1_ < 50% predicted, preoperative renal dysfunction (serum creatinine >1.3 mg/dL), chronic oliguria/anuria requiring dialysis, preoperative laboratory findings of poor liver dysfunction (serum aspartate or alanine amino transferase >100 U/L and serum albumin <2.0 g/dL), preoperative ASA status of 4, history of pancreatitis or current corticosteroid treatment.

There were five different groups of open-heart surgery patients in this study. The groups include: CABG, mitral valve replacement, aortic valve replacement, combined mitral and aortic valve replacement, combined mitral, aortic and/or tricuspid valve replacements.

In our study, we included patients with low and medium risk by evaluating EuroSCORE-related parameters and other possible cardiac risk factors^[9]^. Our goal was to determine the possible risk factors associated with hepatic dysfunction in patients with possible low or medium risk during open-heart surgeries with CPB.

### Anesthesia Procedure

Routine monitoring of cardiac anesthesia was established before anesthesia induction. During anesthesia induction, all patients received intravenous doses of midazolam (Roche, Basel, Switzerland) at a dose of 0.2 mg/kg, fentanyl (Janssen-Cilag, Beerse, Belgium) at a dose of 5 to 10 µg/kg and rocuronium bromide (Organon, Netherlands) at a dose of 0.1 mg/kg. For maintenance, all patients received sevoflurane at an end-tidal concentration of 0.5% to 2% and intravenous maintenance doses of midazolam and fentanyl every half hour. In every operation, cardiac anesthesia-related monitoring devices were applied to all patients and include: application of lead II and V_5_ (anterior axillary line at the same horizontal level as V_4_) electrocardiogram, insertion of a radial arterial peripheral line, use of a pulse oximeter probe on patients’ finger, use of end-tidal carbon dioxide, measurement of nasopharyngeal and rectal temperatures and urine output via Foley catheter, placement of a central venous pressure line, and measurement of pulmonary artery pressures with the placement of a pulmonary artery catheter in some patients, where there is a possible risk of high pulmonary artery pressures.

### Surgical Procedure

The surgical procedure started with a median sternotomy in all patients. Before the beginning of the CPB, heparin at a dose of 300 IU/kg plus additional doses was administered intravenously to provide an activated clotting time (ACT) value > 450 seconds. In all patients, mild hypothermia at a level of 28 to 32°C was established during CPB. Antegrade and retrograde blood cardioplegia were supplied to each patient through appropriate cannulation. Before CPB, each patient received Ringer’s lactate solution at a dose of 10 mL/h. The extracorporeal circuit was established with Ringer’s solution at a dose of 20 mL/kg, 20% mannitol solution at a dose of 0.5 g/kg, sodium bicarbonate 7.5% ampule at a dose of 1 mL/kg and heparin at a dose of 150 IU/kg. A central venous pressure (CVP) value between 8 and 14 mmHg was maintained either by infusion of Ringer’s lactate and isotonic sodium chloride solutions or intravenous furosemide at a dose of 20 mg was used if necessary. Cardiopulmonary bypass was maintained using a Biomedicus roller pump (Biomedicus, Germany) in all patients. Systemic blood flow during CPB was maintained between 2 to 2.5 L/min/m^2^. Systemic blood pressure was kept between 50 and 80 mmHg during CPB. Arterial blood gas analysis was used to measure partial pressures of oxygen (PaO_2_), carbon dioxide (PaCO_2_), and the pH of an arterial blood sample every 60 minutes to keep the following levels: PaO_2_ >250 mmHg, PaCO_2_ between 35 and 45 mmHg, pH between 7.35 and 7.40, hematocrit value between 22 and 28%, and blood glucose value between 100 and 180 mg/dL.

After rewarming with a 37°C maximum heat-exchanger temperature, CPB was discontinued. Intraoperative ventricular tachyarrhythmias were treated with lidocaine at a dose of 1 to 1.5 mg/kg. The reversal of heparin was achieved with the intravenous use of 1.0 to 1.5 mg of protamine per 100 IU heparin. Inotropic support was considered when the mean arterial pressure (MAP) was <65 mmHg. The use of inotropic support during surgery may include one or more of the following: intravenous dobutamine (between 5 and up to 10 µg/kg/min), adrenaline (between 0.02 and up to 0.15 µg/kg/min) and/or noradrenaline (between 0.2 and up to to 1.3 µg/kg/min). The decision to start an inotropic support medication was considered depending on clinical monitoring parameters such as CVP >14 mmHg or heart rate <70 beats/minute. The use of dopamine at a dose of 2 µg/kg/min was provided at the end of CPB if the urine output amount <2 mL/kg. An intravenous bolus dose of furosemide at a dose of 0.2 mg/kg was added and repeated, if necessary.

After surgery, the hematocrit was maintained at approximately 30% by giving packed red blood cells (PRBCs). The mediastinal tube drainage amount <200 mL/h may indicate postoperative bleeding. Any possible coagulation disorder required the monitoring of the parameters of coagulation, such as: ACT, platelet count, activated partial thromboplastin time, and prothrombin time. Depending on the coagulation laboratory data, necessary medical treatments, including a decision for the administration of intravenous protamine, fresh frozen plasma or other blood products were determined. After surgery, blood products, including PRBCs, were given when hemoglobin value <9 g/dL and hematocrit value <25%. During the transfusion of blood products, previous protocols were followed^[8]^.

After completion of the surgical procedure, all patients were transferred to the intensive care unit (ICU) and required mechanical ventilation during the first 24 hours after the operation. During mechanical ventilation, the following settings were considered: pressure-regulated volume control mode, tidal volume of 10 mL/kg, respiratory rate of 14 to 16 breaths per minute, positive end-expiratory pressure of 5 cmH_2_O, and fraction of inspired oxygen of 100%. The determination parameters for adequate mechanical ventilation were based on arterial blood gas measurements to achieve adequate oxygenation and ventilation (pH 7.35-7.45, PaO_2_ >90 mmHg, PaCO_2_ 35-45 mmHg, peripheral oxygen saturation >94%). Discontinuation of mechanical ventilation requires hemodynamic stability, patient awake, a spontaneous breathing continuous positive airway pressure trial, and partial arterial oxygen saturation >80 mmHg on a 40% fraction of inspired oxygen to maintain peripheral capillary oxygen saturation >94%.

### Collected Parameters

In the preoperative period, demographic data of all patients, including gender, age, height, weight, body mass index, ASA classification status, EF and smoking were collected. Possible cardiac risk factors include hypertension, diabetes mellitus, peripheral arterial disease, cerebrovascular disease, COPD, obesity (body mass index >30kg/m^2^), EF and EuroSCORE. Type of operation, CPB and aortic cross-clamping (ACC) times, administration of inotropic agents, number of transfusion of blood products and the use of intra-aortic balloon pump (IABP) were recorded. After the operation, other collected parameters included duration of mechanical ventilation, estimated blood loss, amount of blood drainage, transfusion of PRBCs and fresh frozen plasma in units. After the operation, all patients were followed up until discharge. During this period, ICU stay, in-hospital stay and all adverse events were recorded. The 30-day in-hospital mortality was recorded. After the operation, laboratory data were collected on postoperative days 1, 3, and 7. The collected values include serum albumin, alkaline phosphatase (ALP), alanine transaminase (ALT), aspartate transaminase (AST), lactate dehydrogenase (LDH), total bilirubin (TBil), and gamma-glutamyl transpeptidase (GGT) levels. Preoperatively, all patients had baseline laboratory data collected from peripheral venipuncture. After the operation, blood samples were obtained from the central venous line or from venipuncture. Blood samples were analyzed by the laboratory biochemical analyzer, periodically calibrated and controlled for quality by the clinical laboratory of this institution.

### Primary and Secondary Endpoints

The primary endpoint was the occurrence of a serum total bilirubin concentration >34 µmol/L (2 mg/dL) in any measurement in the postoperative period, and patients were divided into two groups as with or without hyperbilirubinemia depending on this value. Serum total bilirubin levels, as well as other hepatic function tests, were collected preoperatively and postoperatively on days 1, 3 and 7. The collected parameters were compared within the groups and changes in preoperative values were evaluated. The secondary endpoint was the effect of hyperbilirubinemia on various possible clinical risk factors such as: ACC time; CPB time; use of inotropic support; use of IABP; prolonged mechanical ventilation; development of pneumonia; perioperative myocardial infarction; cerebrovascular event (stroke, transient ischemic attack), seizure; atrial fibrillation and other rhythm disorders; need for renal replacement therapy; reoperation for bleeding; ICU stay; hospital stay; and other adverse events such as development of sepsis or need for tracheostomy^[9,10]^.

### Statistical Analysis

SPSS software for Windows version 17.0 (Statistical Package for the Social Sciences Inc., Chicago, IL, USA) was used. The normality of the study data was tested utilizing the one-sample Kolmogorov-Smirnov test. Continuous variables were expressed as median (minimum-maximum). As a result of the analysis, non-parametric test of Mann-Whitney U test was used for comparison of two independent continuous groups. Categorical variables were expressed as numbers and percentages. For categorical parameters, chi-square analysis and Fisher’s exact test were used when appropriate. The trial size is determined depending on previous published data^[1,17]^. In the study by Wang et al., the sample size calculation to include 302 patients into the study was determined by evaluating a statiscally significant difference in mortality (5.6% *vs*. 0.5%, *P*<0.01) between the group of patients with or without hyperbilirubinemia^[1]^. The serum values of various well-known hepatic dysfunction related parameters were collected at different times: preoperatively and on postoperative days 1, 3 and 7 and these values were compared using two-way analysis of variance (ANOVA) for repeated measures. Post hoc analysis was performed when appropriate. Operative variables, including CPB time, ACC time, surgical procedure type, amount of blood components transfused, extubation time, length of ICU stay and hospital stay and other possible risk factors were evaluated with a multivariate regression analysis model to identify predictors for developing postoperative hyperbilirubinemia by a forward stepwise (conditional) procedure. Possible risk factors were investigated and calculated using Cox regression analysis^[11]^.

## RESULTS

A total of 378 patients were enrolled into the study. Seventy-one patients were excluded for possible high cardiac risk factors and the study was performed on 307 patients. In this group of patients, the surgical types performed were CABG (n=176) and valve repair surgery (mitral, mitral and aortic valve and/or tricuspid valve) (n=131). Depending on the methods and definition of hyperbilirubinemia in the postoperative period, patients were divided into groups with or without postoperative hyperbilirubinemia.

Preoperative demographic data, preoperative comorbidities, possible factors related to cardiac risk, EuroSCORE and distribution of surgical operations are presented for both groups of patients (with and without hyperbilirubinemia) in Table 1. There was no statistically significant difference between age, gender, height, weight and body mass index (BMI) between patients with or without hyperbilirubinemia (*P*>0.05). During a 10-day period after cardiac surgery, patients were investigated with laboratory findings for possible development of hepatic dysfunction. All patients were divided into two groups with or without hyperbilirubinemia, and this was defined by the occurrence of a plasma TBil >34 µmol/L (2 mg/dL) in any measurement during the postoperative period. Hyperbilirubinemia developed in 18 (5.86%) of the 307 patients. Therefore, the overall incidence of postoperative jaundice was 5.86% in our patient population. Postoperative hyperbilirubinemia was observed in 7 of 176 patients (4%) who underwent CABG and in 11 of 131 patients (8.4%) who underwent valve replacement surgeries. In comparison to CABG procedures (n=176), postoperative hyperbilirubinemia was observed more frequently in patients undergoing valve surgeries (n=131) (*P*=0.027), and this is shown in Table 1.

**Table 1:** Comparison of preoperative demographic data, comorbidities, and distribution of surgical operations in patients with or without hyperbilirubinemia undergoing open-heart surgeries.

	Without hyperbilirubinemia (n=289)				With hyperbilirubinemia (n=18)			*P*-value
	n (%)	Median		Quartile range	n (%)	Median	Quartile range	
Gender								
Male	196 (68)				14 (78)			0.378
Female	93 (32)				4 (22)			
Age (years)		58		22-79		50	47-76	0.221
Height (cm)		1.65		1.38-1.85		1.65	1.4-1.8	0.262
Weight (kg)		75		50-127		74.5	61-98	0.185
Body mass index (kg/m^2^)		27.92		18.31-45		29.5	23.8-36	0.625
Preoperative comorbidities								
Hypertension	131 (45)				11 (61)			0.193
Diabetes mellitus	81 (28)				7 (39)			0.323
Obesity	57 (20)				7 (39)			0.052
Smoking	88 (30)				3 (17)			0.214
Peripheral artery disease	39 (14)				3 (17)			0.704
Cerebrovascular disease	26 (9)				4 (22)			0.071
COPD	45 (16)				5 (28)			0.174
Congestive heart failure	20 (7)				4 (22)			0.019
History of AMI (<90 days)	34 (12)				3 (17)			0.535
EF (%)		55	30-65			40	30-50	<0.001
EuroSCORE		2.0	0-7.0			4.0	2.0-6.0	0.024
Distribution of surgical operations (n,%)								
CABG	169 (59)				7 (39)			0.027
Mitral valve	35 (12)				2 (11)			
Aortic valve	30 (10)				0 (0)			
Mitral and aortic valve	27 (9)				4 (22)			
Mitral, aortic and/or tricuspid valve	28 (10)				5 (28)			

*P value <0.05 statistically significant; Mann-Whitney U test; Chi-square test; n(%)=number (percentage). AMI=acute myocardial infarction; COPD=chronic obstructive pulmonary disease; CABG=coronary artery bypass graft surgery;EF=ejection fraction

Patients with hyperbilirubinemia tend to have a BMI >30 (obesity) compared with patients without hyperbilirubinemia (*P*=0.052). In addition, EF in patients with hyperbilirubinemia (40% [30-50%]) is lower than in patients without hyperbilirubinemia (55% [30-65%]) (*P*<0.001). There were no significant differences in the number of patients with a history of acute myocardial infarction (*P*=0.535). However, in comparison between EuroSCORE values, patients with hyperbilirubinemia had higher EuroSCORE values (4.0 [2.0-6.0]) in comparison to patients without hyperbilirubinemia (2.0 [0-7.0]) (*P*=0.024). A EuroSCORE of 6 was found in 20 (7%) patients without hyperbilirubinemia and in 2 (11%) patients with hyperbilirubinemia, whereas a EuroSCORE of 7 was observed in 10 (4%) patients without hyperbilirubinemia and in none of the patients with hyperbilirubinemia.

The comparison of hepatic dysfunction parameters in patients who underwent CABG and valve surgeries is presented in Table 2. It is noted that total bilirubin values were significantly different between CABG and valve surgery patients preoperatively (*P*=0.025). The repeated measure analysis of total bilirubin values shows significant differences within CABG group and within valve surgery group (*P*=0.002 and *P*=0.001, respectively). For other hepatic parameters, comparisons between CABG and valve surgery groups showed no significant differences at different collection times (*P*>0.05, Table 2).

**Table 2:** Comparison between groups and within groups of hepatic dysfunction parameters in patients with coronary artery bypass graft (CABG) and valve surgeries.

Parameters	Diagnosis	Preoperative	Day 1	Day 3	Day 7	*P*-value*
ALP (IU/L)	CABG	74 (28-320)	66 (31-172)	75 (15-203)	76 (30-1011)	0.510
	Valve surgery	72 (42-320)	64 (42-112)	67 (36-203)	76 (30-200)	0.498
	*P*-value	0.575	0.298	0.119	0.430	
AST (IU/L)	CABG	22 (13-84)	45 (16-264)	43 (17-381)	35 (14-214)	0.553
	Valve surgery	22 (15-83)	45 (17-473)	39 (16-140)	34 (14-156)	0.474
	*P*-value	0.747	0.186	0.256	0.991	
ALT (IU/L)	CABG	19 (7-86)	45 (13-144)	37 (10-148)	33 (10-188)	0.617
	Valve surgery	20 (7-86)	45 (24-144)	37 (15-147)	33 (12-178)	0.462
	*P*-value	0.229	0.598	0.507	0.459	
GGT (IU/L)	CABG	28 (7-179)	30 (8-147)	43 (10-308)	34 (11-391)	0.281
	Valve surgery	29 (7-107)	29 (8-90)	36 (10-239)	34 (11-391)	0.234
	*P*-value	0.632	0.338	0.634	0.701	
LDH (U/L)	CABG	358 (319-722)	1139 (679-2119)	1107 (625-1983)	678 (612-1654)	0.768
	Valve surgery	395 (155-1577)	757 (309-2155)	674 (101-2001)	600 (250-1909)	0.781
	*P*-value	0.067	0.018	0.003	0.01	
TBil (mg/dL)	CABG	0.55 (0.25-1.90)	0.70 (0.40-2.20)	0.70 (0.40-2.80)	0.80 (0.30-2.80)	0.002
	Valve surgery	0.55 (0.25-1.40)	0.70 (0.40-2.20)	0.70 (0.40-2.60)	0.80 (0.30-2.10)	0.001
	*P*-value	0.025	0.878	0.520	0.883	
Albumin (mg/dL)	CABG	4.1 (2.4-5.6)	3.6 (2.5-4.8)	3.4 (2.4-4.7)	3.6 (2.6-4.8)	0.248
	Valve surgery	4.2 (3.2-5.6)	3.7 (2.5-4.2)	3.5 (2.6-3.9)	3.6 (2.6-4.1)	0.271
	*P*-value	0.469	0.602	0.431	0.439	

* *P*-value <0.05 statistically significant; two-way ANOVA. ALP=alkaline phosphatase; ALT=alanine aminotransferase; AST=aspartate aminotransferase; CABG=coronary artery bypass graft; GGT=gamma glutamyl tranpeptidase; LDH=lactic dehydrogenase; TBil=total bilirubin

The most relevant intraoperative and postoperative parameters were evaluated among patients with hyperbilirubinemia (n=18) and without hyperbilirubinemia (n=289) in Table 3. The comparison showed that CPB time, ACC time, extubation time, ICU stay and hospital stay are significantly prolonged in patients with hyperbilirubinemia and show a significant difference between groups (*P*<0.001, for each parameter). IABP usage was recorded in 3 patients with hyperbilirubinemia (3/18, 16.7%) and in 10 patients without hyperbilirubinemia (10/289, 3.5%) (*P*=0.007). The use of PRBCs intraoperatively and postoperatively in the first 24 hours was recorded for patients with or without hyperbilirubinemia, and the comparison between CABG and valve surgeries is shown in Table 3 (*P*<0.001 and *P*<0.001, respectively).

**Table 3:** Comparison of pertinent intraoperative and postoperative parameters with or without hyperbilirubinemia

	Without hyperbilirubinemia (n=289)		With hyperbilirubinemia n=18)		*P*-value
	Median	Quartile range	Median	Quartile range	
CPB time (min)	99	40-325	139	102-212	<0.001
ACC time (min)	56	16-207	112	77-166	<0.001
Extubation time (hours)	9	4-28	20	8-28	<0.001
Mean intensive care stay (days)	2	1-13	7	4-10	<0.001
Hospital stay (days)	14	9-25	24.5	13-42	<0.001
Mortality, n (%)	3 (1)		0 (0)		0.664

*P-value <0.05 statistically significant; median (minimum-maximum); Mann-Whitney U test; min: minute; n= number; %=percentage ACC=aortic cross-clamp; CPB=cardiopulmonary bypass

Later, we performed a multiple logistic regression analysis of the risk factors for hyperbilirubinemia (Table 4). The independent risk factors for early postoperative hyperbilirubinemia are: EF (OR 0.797, *P*=0.03, 95% CI 0.649-0.978); ACC time (OR 1.101, *P*=0.014, 95% CI 1.020-1.190); ICU stay (OR 1.792, *P*=0.001, 95% CI 1.255-2.558); and extubation time (OR 1.154, *P*=0.045, 95% CI 1.003-1.327).

**Table 4:** Multiple logistic regression analysis of risk factors for postoperative hyperbilirubinemia in patients undergoing open-heart surgery.

	B	SE	*P*-value*	Odds ratio	95% CI for EXP(B)	
					Lower	Upper
EF	-0.227	0.105	0.030	0.797	0.649	0.978
ACC time	0.097	0.039	0.014	1.101	1.020	1.190
CPB time	-0.028	0.023	0.222	0.973	0.931	1.017
IABP	-0.556	1.534	0.717	0.573	0.028	11.589
ICU stay	0.583	0.182	0.001	1.792	1.255	2.558
Extubation time	0.143	0.071	0.045	1.154	1.003	1.327
Constant	-3.609	3.877	0.352	0.027		

* *P*-value <0.05 statistically significant; B=regression coefficient; SE=standard error. ACC=aortic cross-clamp; CI=confidence interval; CPB=cardiopulmonary bypass; EF=ejection fraction; IABP=intra-aortic balloon pump; ICU=intensive care unit

**Fig. 1:**
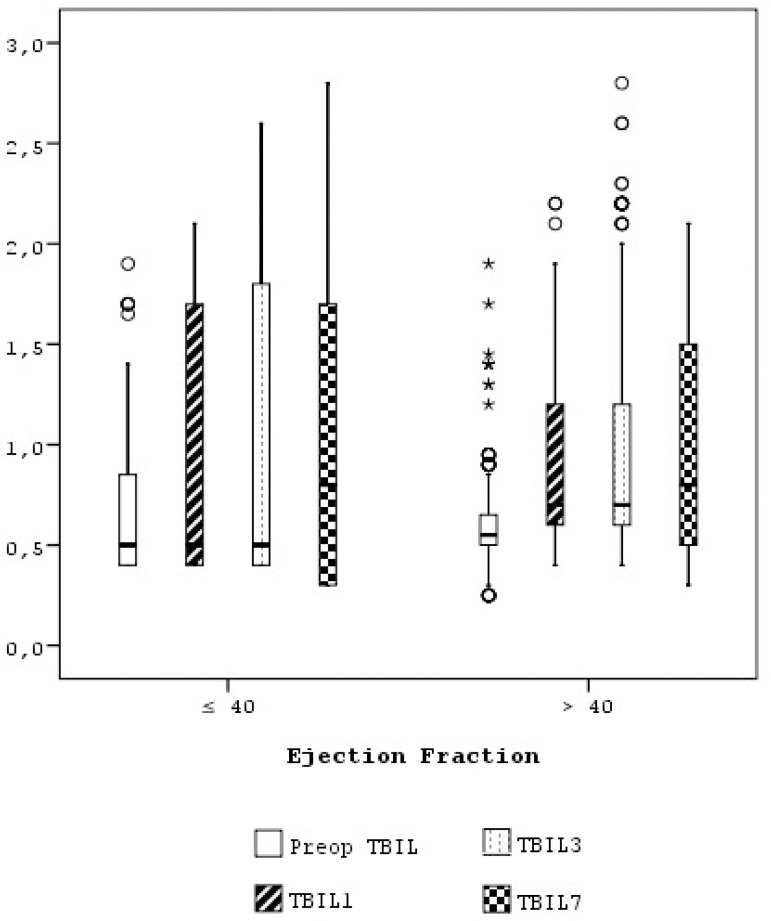
Comparison of box plot values of total bilirubin on collected time points depending on an EF of 40% as the determination point (total bilirubin values on collected time points were significantly higher for patients with an EF ≤40% in comparison to patients with EF >40%). TBil=total bilirubin.

**Fig. 2:**
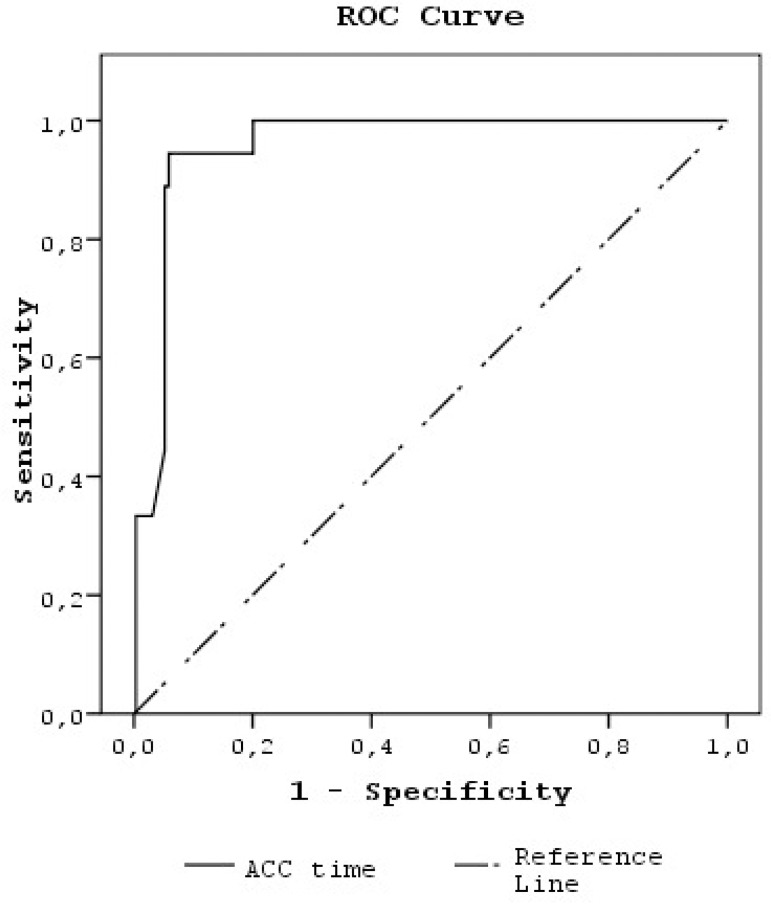
The receiver operating characteristic (ROC) curve is used to determine the optimal cutoff value of the ACC time for predicting the development of postoperative hyperbilirubinemia during open-heart surgeries. An ACC time threshold value of 102.5 minutes was able to discriminate the development of postoperative hyperbilirubinemia in patients undergoing open-heart surgery with a sensitivity of 94.4% and a specificity of 94.2% (area under the curve: 0.96±0.01). ACC=aortic cross-clamp.

The comparison of box plot values of TBil on collected time points; depending on the EF of 40% as the determination point; is shown in Figure 1. This box plot statistical analysis shows that total bilirubin values on collected time points were significantly higher for patients with an EF ≤40% in comparison to patients with an EF >40%. In Figure 2, a receiver operating characteristic (ROC) curve is used and an ACC time threshold value of 102.5 minutes was able to discriminate the development of postoperative hyperbilirubinemia in patients undergoing open-heart surgery with a sensitivity of 94.4% and a specificity of 94.2% (area under the curve: 0.96±0.01). The elevated serum bilirubin level (presence of hyperbilirubinemia) was correlated with preoperative EF (r=-0.264, *P*<0.001), operative ACC time (r=0.372, *P*<0.001), operative CPB time (r=0.328, *P*<0.001), IABP usage (r=0.154, *P*=0.007), and postoperative parameters such as multiple valve surgeries (r=0.137, *P*=0.016), development of pneumonia (r=1.000, *P*<0.001), renal failure (r=0.773, *P*<0.001), intraoperative and postoperative 24-hour blood transfusions (r=0.407, *P*<0.001), noradrenaline infusion (r=0.745, *P*<0.001), atrial arrhythmia (r=0.120, *P*=0.036), ventricular arrhythmia (r=0.712, *P*<0.001), extubation time (r=0.314, *P*<0.001), ICU stay (r=0.428, *P*<0.001) and hospital stay (r=0.377, *P*<0.001).

## DISCUSSION

The risk factors that may contribute to hepatic dysfunction after open-heart surgery with CPB have been investigated previously^[2,5-7]^. A recent study provides data on the causes of postoperative hepatic dysfunction after cardiac surgeries^[11]^. The main factors that cause hepatic dysfunction include: decreased hepatic blood flow, prolonged CPB duration, ACC times, infection, drugs, anesthetic agents, inflammatory cytokines and some other risk factors, such as older age or a poor nutritional status^[12-17]^. Many risk factors have been associated with hyperbilirubinemia and risk factors that increase mortality also cause postoperative dysfunction^[9,11-13]^. For these reasons, we hypothesized to perform our study at a patient group with low and moderate risk factors for postoperative mortality. During the enrollment in the study, we excluded the possible high-risk factors that increase postoperative mortality and, for this purpose, we referred to the EuroSCORE cardiac risk scoring system^[9]^. In our study, before inclusion in the study, exclusion criteria to prevent patients with high EuroSCORE and patients with possible high cardiac risks were applied. However, some patients with a EuroSCORE of 6 and 7 were also included into the study. Overall, 32 (11%) of the 307 patients with a EuroSCORE of 6 and 7 were included in our patient group. EuroSCORE ≥6 was not an exclusion criterion. Therefore, we have included these patients. In our opinion, as our study group contains patients with low or moderate cardiac risk, the overall incidence of postoperative jaundice was 5.86% in our patient population. In the literature, the incidence of postoperative hyperbilirubinemia was reported in a wide range, and some of the reported percentages include 3.2%, 8.6%, 19% and up to 26.5%^[1,2,4,16]^. In a recent study, postoperative hyperbilirubinemia was reported as 19% and the univariate analysis of risk factors for hyperbilirubinemia shows that preoperative risk factors, including cerebrovascular disease, renal dysfunction, liver dysfunction, infective endocarditis and history of heart failure have a statistically significant correlation with postoperative hyperbilirubinemia^[18]^.

In our study, the main objective was to determine the risk factors associated with hepatic dysfunction in patients undergoing open-heart surgery with CPB and our results show that the independent risk factors of early postoperative hyperbilirubinemia are: EF, ACC time, ICU stay, and extubation time. In previous studies, the blood volume in liver arteries has been reported to decrease by 20 to 25% during CPB, and ACC time is crucial to prevent hypoxia-related hepatic dysfunction and liver failure^[11-13]^. In addition, some studies point out the importance of cardiac output state and the use of inotropic agents during open-heart surgeries^[18-20]^. These discussions support our findings of EF and ACC time as independent risk factors. It was reported that patients with hepatic dysfunction in the early postoperative period after open-heart surgeries with CPB had a longer ICU stay with increased risk of morbidities such as prolonged mechanical ventilation, increased risk of pneumonia, sepsis and mortality^[10,18,21,22]^. These reports support our findings that ICU stay and extubation time are important risk factors of early postoperative hyperbilirubinemia.

The incidence of hyperbilirubinemia has increased in patients undergoing multiple valvular replacement surgeries, and the reasons include different valvular technologies, prolonged duration of CPB time during two or more valve replacements, difficulties in performance during these procedures, decreased hepatic blood flow, and advanced heart failure state^[1,4,5,14,23]^. In our study, valve surgeries has been found to cause more postoperative hyperbilirubinemia in comparison to CABG surgeries and our study supports the previous reports. Other frequently reported factors include increased blood transfusions and prolonged CPB duration.

Other important independent risk factors for postoperative hyperbilirubinemia are low EF and prolonged ACC time^[12,14,16]^. Our findings show a correlation between postoperative elevated serum bilirubin in the first seven days and preoperative EF, presence of multiple valve replacement procedures, intraoperative ACC and CPB times, extubation time, ICU stay, intraoperative and 24-hour postoperative blood transfusions, noradrenaline inotropic usage, IABP usage (*P*<0.05). These findings are also presented in previous studies, showing that various parameters show significant correlations with postoperative hyperbilirubinemia^[5,12,14,16,22]^. Patients with severe preoperative cardiac failure may have higher right atrial pressure and ascites and, with a congested liver, their capacity to dispose the bilirubin load may be impaired^[1,4,5,23,24]^.

One of the limitations of our study is the lack of determination of serum levels of direct and indirect bilirubin values. These values have been used in the literature to predict the occurrence of an increase in bilirubin load resulting from hemolysis (unconjugated, indirect bilirubin). Hemolysis from CPB, cardiotomy suction and mechanical prosthesis are responsible for a higher incidence and greater severity of postoperative hyperbilirubinemia. Other factors, such as the large volume of transfused blood, may be related to large loads of bilirubin, mainly conjugated; direct bilirubin may be impaired^[1]^.

In our study, we showed that another factor in the development of severe hepatic dysfunction is the number of blood transfusions during cardiac operations. Heart valve surgeries, low EF, low cardiac output, hypotension, hypoxia, or hypothermia have been reported to be associated with hyperbilirubinemia and inability to respond appropriately to the bilirubin load caused by an excessive amount of blood transfusions during open-heart surgeries with CPB^[4,5,14,15,22-24]^. There are other data that support these findings. During cardiac surgeries, especially in the intraoperative stage, hypovolemia, prolonged CPB time and ACC time and administration of inotropic agents may cause hypoperfusion^[11-15]^. In two other studies, it was reported that, in patients with right heart failure and tricuspid regurgitation, the incidence of postoperative hyperbilirubinemia was significantly higher^[5,15]^.

Michalopoulos et al.^[2]^support that hepatic dysfunction follows other intraoperative and postoperative complications, such as low cardiac output syndrome, which requires administration of inotropic agents and the use of IABP during open-heart surgeries with CPB. In our study, 13 patients required intraoperative cardiac support with IABP, and IABP usage is higher in patients with hyperbilirubinemia in comparison to patients without hyperbilirubinemia (16.7% *vs*. 3.5%). It has also been reported that there is a relationship between hyperbilirubinemia and a higher rate of postoperative infection and, subsequently, this inflammatory process leads to the development of postoperative adverse events and is associated with prolonged mechanical ventilation and ICU stay^[15,20-22]^.

In a recent study, from a total of 12556 patients in a 10-year period, although all patients with end-stage liver disease were included in the study, only 1272 (10.1) of the patients developed hyperbilirubinemia, defined as bilirubin concentration >3 mg/dL^[11]^. In our study, the overall incidence of hyperbilirubinemia is lower than the previously published data, however, we have tried to exclude many risk factors that are associated with increased mortality and morbidity and, for this purpose, we used EuroSCORE scoring system and enrolled patients with a low and moderate cardiac risk in our study^[9]^.

### Limitations of the study

Our study has several limitations. Our data were collected prospectively, however, some data might be missing, such as information on the presence of viral or drug-induced hepatitis and hepatic dysfunction related to anesthesia has not been determined. There is a lack of evaluation of serum levels of direct and indirect bilirubin values to determine conjugated and unconjugated hyperbilirubinemia. The 30-day mortality was recorded, however, a long-term analysis of the postoperative outcome was not performed. We hope our results may help to identify important risk factors for hyperbilirubinemia.

## CONCLUSION

Independent risk factors for early postoperative hyperbilirubinemia were found, as EF, ACC time, ICU stay and extubation time. Patients undergoing valve replacement procedures are at a higher risk for the development of postoperative hyperbilirubinemia in comparison to CABG procedures. Other important parameters that show a correlation with hyperbilirubinemia include intraoperative and 24-hour postoperative blood transfusions, noradrenaline inotropic usage, and IABP usage.

**Table:** 

Authors' roles & responsibilities	
AB	Substantial contributions to the conception or design of the work; analysis, or interpretation of data for the work; drafting the work or revising it critically for important intellectual content; data collection; final approval of the version to be published
GS	Substantial contributions to the conception or design of the work; analysis, or interpretation of data for the work; drafting the work or revising it critically for important intellectual content; final approval of the version to be published
MD	Substantial contributions to the conception or design of the work; analysis, or interpretation of data for the work; statistics; data collection; final approval of the version to be published
IO	Substantial contributions to the conception or design of the work; analysis, or interpretation of data for the work; statistics; data collection; final approval of the version to be published
